# RNA Splicing: A New Player in the DNA Damage Response

**DOI:** 10.1155/2013/153634

**Published:** 2013-09-12

**Authors:** Silvia C. Lenzken, Alessia Loffreda, Silvia M. L. Barabino

**Affiliations:** Department of Biotechnology and Biosciences, University of Milano-Bicocca, Piazza della Scienza 2, I-20126 Milan, Italy

## Abstract

It is widely accepted that tumorigenesis is a multistep process characterized by the sequential accumulation of genetic alterations. However, the molecular basis of genomic instability in cancer is still partially understood. The observation that hereditary cancers are often characterized by mutations in DNA repair and checkpoint genes suggests that accumulation of DNA damage is a major contributor to the oncogenic transformation. It is therefore of great interest to identify all the cellular pathways that contribute to the response to DNA damage. Recently, RNA processing has emerged as a novel pathway that may contribute to the maintenance of genome stability. In this review, we illustrate several different mechanisms through which pre-mRNA splicing and genomic stability can influence each other. We specifically focus on the role of splicing factors in the DNA damage response and describe how, in turn, activation of the DDR can influence the activity of splicing factors.

## 1. Overview of the DNA Damage Response

Genomic instability is one of the most common characteristics of tumor cells and is probably due to the combined effect of DNA damage, tumor-specific DNA repair defects, and a failure to arrest the cell cycle before the damaged DNA is passed on to daughter cells. Genomic instability is recognized as a characteristic of most solid tumors and adult-onset leukaemias and is manifested as alterations in chromosome number and structure (chromosomal instability) and as changes to the structure of DNA, such as nucleotide substitutions, insertions, and deletions. To maintain genomic stability and to counteract DNA damage, cells have evolved a complex cellular response, called DNA damage response (DDR), which is coordinated by the DNA damage checkpoints [1, 2]. 

Somatic mutations in DDR genes have been found in several cancer types [3]. Indeed, on one hand, inactivation of the DDR favors the accumulation of mutations in proto-oncogenes increasing the risk of tumor development. On the other, since the anticancer activity of most chemotherapeutic drugs relies on the induction of DNA damage, alterations in the DDR also affect the tumor's sensitivity to chemotherapy [4].

Conceptually the molecules that orchestrate the DDR can be functionally organized in sensors, mediators, transducers and effectors. Recognition of DNA damage is the first step in the activation of the signaling cascade that controls the DNA damage checkpoints. DNA lesions are recognized by various sensor proteins: the MRN (MRE11-RAD50-NBS1) complex that signals double-strand DNA breaks (DBSs), and by RPA that binds single-strand DNA at sites of DNA damage. Subsequently, recruitment and activation of the highly conserved apical DDR kinases ataxia-telangiectasia mutated (ATM) and ataxia-telangiectasia and RAD3-related (ATR) occur. In both yeast and mammals Tel1/ATM recognizes DSBs, while Mec1/ATR is activated in the presence of long single-stranded DNA tracts. Once activated, ATR and ATM transduce the DDR signal by promoting the phosphorylation/activation of downstream kinases, such as CHK1 and CHK2, which in turn regulate downstream checkpoint effectors. Checkpoint activation elicits a multifaceted cellular response that coordinates cell cycle progression with DNA repair activity, thus allowing cells to block the cell cycle until the damage is repaired. In addition, ATM phosphorylates the histone 2A variant *γ*H2AX that marks chromatin regions flanking DSBs. These phosphorylation events promote the recruitment of several mediator proteins that facilitate ATM/ATR signaling (see [5]).

Recently, a novel layer of complexity in the cellular response to DNA damage has emerged with the involvement of RNA metabolism. A first link between mRNA biogenesis and genome stability has been provided by the observation that transcription is inhibited in response to DNA damage, both generally and locally at DNA damage sites [6, 7]. Changes in the pre-mRNA splicing pattern of crucial genes in the DDR have long been observed (for review see [8]), and splicing factors have been observed to change their intracellular distribution following genotoxic damage. Finally, DNA damage is known to affect mRNA stability both positively and negatively (for review see [9]). This review is focused on the interplay between AS and DDR. We initially discuss how activation of the DDR signaling cascade can influence the activity of splicing factors and how this can affect cell fate. Then, we examine how, in turn, alternative splicing factors can contribute to the DDR. Finally, we will discuss how alternative splicing regulators can represent novel targets for cancer therapies.

## 2. How DDR Can Affect Alternative Pre-mRNA Splicing (AS)

In recent years several reports have uncovered how DNA damage can induce splicing changes that give rise to mRNA variants encoding different protein isoforms with the potential to affect the cellular response and the cell fate. A first indication that the DDR can alter AS came from the observation that *Drosophila* S2 cells treated with camptothecin, a topoisomerase I inhibitor that leads to replication forks arrest, or exposed to ionizing radiation (IR) express specific alternative splicing variants of the transcription factor TAF1 that was shown to control the G2/M transition [10]. Alternative splicing of TAF1 in response to DNA damage was shown to depend on ATM/CHK2 and ATR/CHK1 signaling and to induce degradation of the splicing factor Tra2 [10, 11]. Another example is provided by the effect of UV irradiation and cisplatin on the splicing of MDM2 and MDM4 transcripts [12]. The MDM2 gene encodes an E3 ubiquitin ligase that targets p53 for proteasome-mediated degradation. MDM2 expression is positively controlled by p53 at the transcription level, generating a feedback loop. The proteins encoded by the mRNA variants induced by DNA damage lack the p53 interaction domain so that they may favor p53 activation [12]. A detailed description of the regulation of the alternative splicing of the MDM2 transcript in response to various genotoxic treatments can be found in [8].

**Figure 1:**
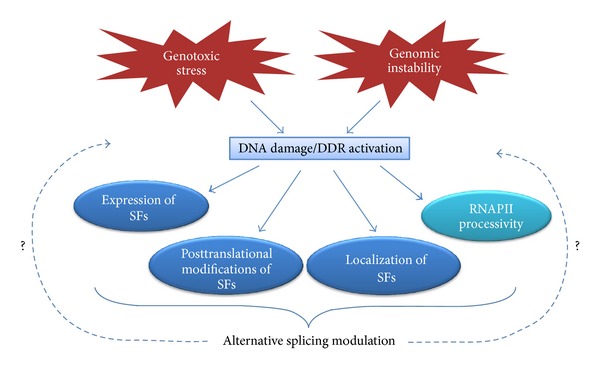
How the DDR can affect alternative splicing. The activation of the DDR can modify alternative splicing by affecting the expression, or by inducing posttranslational modifications of splicing factors (SFs), that may alter their intracellular localization and/or their activity. Moreover, also the elongation activity of RNA polymerase II (RNAPII) can be influenced by genotoxic stress, modifying in turn pre-mRNA splicing.

How genotoxic stress can influence the activity of the splicing machinery is to date largely unknown. Two major mechanisms are known to control the activity of splicing factors in response to external and internal stimuli: (i) changes in expression level and (ii) posttranslational modifications. In addition, it has emerged in recent years that pre-mRNA splicing occurs largely cotranscriptionally and that also the processivity of RNA polymerase II (RNAPII) can influence the recognition of alternative exons ([13, 14], Figure 1). 

### 2.1. The Expression Level of Splicing Factors Changes in Response to DNA Damage

The simplest way by which DNA damage can affect the splicing machinery is by modifying the expression level of specific splicing factors. SR proteins and hnRNP proteins were the first splicing factors identified [15, 16]. These proteins are components of the basal splicing machinery. However, since their concentration can influence splice site selection, they contribute to alternative splicing. Indeed, deregulation of several members of the SR protein and hnRNP families of splicing regulators has been observed following various genotoxic treatments [17, 18]. One additional recent example is provided by the upregulation of SC35 by E2F1, a transcription factor that has a key function during S phase progression and apoptosis in response to DNA-damaging agents [19].

### 2.2. Posttranslational Modification of Splicing Factors

As mentioned above the presence of phosphorylated *γ*H2AX is a hallmark of sites of DSBs. However, phosphorylation of H2AX is only the tip of the iceberg: a host of posttranslational modifications of both the histones and components of the DDR machinery have been reported at sites of DNA damage, including acetylation, ubiquitination and SUMOylation, and arginine methylation (for review see [20]). These modifications play a central role in coordinating cell cycle progression and DNA repair [21]. Therefore, it is not surprising that activation of the damage signaling cascade can lead to the post-translational modification of splicing factors that can modify their intracellular localization and/or activity. Here, we will discuss examples of posttranslational modifications of splicing regulatory proteins in response to DNA damage that have recently been reported.

#### 2.2.1. Phosphorylation

The activity and the intracellular distribution of the serine/arginine (SR) proteins are tightly regulated by phosphorylation. SR proteins are RNA-binding proteins that are characterized by at least one domain enriched in RS dipeptides. This region, often located at the C-terminus of the protein, is termed RS domain and is subjected to reversible phosphorylation. The assembly of the splicing complex and the catalysis of the splicing reaction require dephosphorylation/phosphorylation cycles. While phosphorylated SR proteins favor spliceosome assembly, intron removal is associated with dephosphorylation of SR proteins. After splicing, a subset of SR proteins remains associated with the mature mRNA and is exported to the cytoplasm. Here, their reimport into the nucleus requires phosphorylation by SRPK1 and SRPK2, two SR protein kinases that are predominantly localized in the cytoplasm. It was recently shown that genotoxic stress can induce the phosphorylation and the relocalization of these kinases to the nucleus where they in turn hyperphosphorylate SR proteins leading to changes in pre-mRNA splicing [22, 23]. Moreover, chronic replication-dependent DNA damage was shown to induce the hyperphosphorylation of ASF/SF2 (SRSF1) [24].

Several studies underline the importance for catalysis of dephosphorylation within the assembled spliceosome [25–27]. Interestingly, the protein phosphatase PPM1G, which promotes pre-mRNA splicing, is phosphorylated in response to DNA damage and is rapidly recruited to DNA damage sites [28].

#### 2.2.2. Acetylation

The first evidence of the role of lysine acetylation in the DDR came with the observation that overexpression of a dominant-negative allele of the acetyltransferase TIP60 reduced the efficiency of DSB repair [29]. Now, we know several TIP60 targets among crucial DDR factors, including H2AX, H4, and ATM [30]. A recent quantitative mass spectrometry analysis revealed that splicing factors, including SR proteins and hnRNPs, have numerous acetylation sites [31], often located within their RNA-binding domain. Since acetylation of lysine neutralizes the positive charge of the amino acid, this reversible modification could contribute to the regulation of their activity in splicing. Consistent with this report, Edmond and colleagues recently identified the SR protein ASF/SF2 as a novel substrate of TIP60 acetyltransferase activity in response to genotoxic treatments [23]. In the case of ASF/SF2, however, acetylation does not influence the splicing activity but rather controls its protein turnover by promoting degradation. Although these observations clearly demonstrate that acetylation can contribute to the regulation of the DDR, a recent genome-wide characterization of the DDR-regulated acetylome revealed a rather weak overall increase in site-specific acetylation compared to phosphorylation suggesting that acetylation may be a more selective/subtle modification [28].

#### 2.2.3. Ubiquitination/SUMOylation

In addition to targeting proteins to proteasome-mediated degradation, ubiquitination has emerged as an important regulatory signal that functions in many cellular processes. Ubiquitination involves the covalent attachment of a 76 amino acid ubiquitin chain to a lysine of the modified protein by an E3 ubiquitin ligase. Multiple lysines can be ubiquitinated in the target protein, and following addition of the first ubiquitin additional ubiquitin molecules can be added, yielding a polyubiquitin chain. 

Many different E3 ubiquitin ligases participate in the DDR. For example, RNF2 catalyzes the monoubiquitination of H2AX contributing to ATM recruitment [32, 33], while depletion of RNF4 impairs RAP80, BRCA1, and RAD51 recruitment to sites of DNA damage [34–36]. 

Indirect evidence suggests that ubiquitination of splicing factors may also be involved in the DDR. Ubiquitin regulates spliceosome assembly [37]. Moreover, the essential yeast splicing factor Prp19 and its human ortholog have E3 ligase activity *in vitro* [38]. A role of PRP19 in the mammalian DDR first emerged with the report that it was strongly upregulated by DNA damage in human cells and that its depletion by siRNA resulted in an accumulation of DSBs and apoptosis and reduced survival after exposure to ionizing radiation [39]. Prp19 is part of a large complex that contains >30 proteins [40]. PRP19/Pso4 itself is modified by ubiquitination in response to DNA damage, and this modification reduces its affinity for other members of the Pso4 complex [41]. 

Besides ubiquitin, vertebrate cells encode several other ubiquitin-like proteins that are structurally related to ubiquitin. The best characterized one is small ubiquitin modifier (SUMO). Similar to ubiquitination, SUMOylation is a reversible posttranslational modification that plays a crucial role in the control of the DDR and in DNA repair pathways by modulating protein:protein interactions (for review see [42]). 

ASF/SF2, the best characterized member of the SR protein family of splicing factors, can act as a cofactor stimulating SUMO conjugation by the SUMO E2 conjugating enzyme Ubc9 [43]. ASF/SF2 also interacts with PIAS1 and regulates its SUMO E3 ligase activity in response to DNA damage [43]. 

HnRNP K, as several other hnRNPs, is SUMOylated [44]. HnRNP K is a multifunctional protein involved in many steps of mRNA biogenesis [45]. Various genotoxic treatments stimulate SUMOylation of hnRNP K by the Polycomb E3 ligase Pc2 that in turn is activated by DNA damage via phosphorylation by HIPK2 kinase [46]. HnRNP K cooperates with p53 in the transcriptional activation of cell cycle arrest genes such as 14-3-3*σ*, GADD45, and p21 in response to DNA damage [47], and its SUMOylation stimulates p53 transcriptional activity [46].

### 2.3. Transcriptional Effects on AS

Many chemotherapeutic drugs are potent inducers of DNA damage that interferes with transcription. Platinum derivatives such as cisplatin and carboplatin induce DNA adducts and intra- and interstrands cross-links between purine bases. Platinated adducts distort the DNA helix impairing replication and transcription elongation, which in turn can lead to the formation of DSBs [48]. Camptothecin (CPT) is a topoisomerase I inhibitor that leads to a block of transcription elongation and DNA replication. Doxorubicin is an inhibitor of DNA topoisomerase II that induces structural alterations in promoter DNA [49].

Although pre-mRNA splicing can occur independently of transcription, many different studies have provided evidence that AS, as other RNA processing events required for the synthesis of the mature mRNA, is mostly cotranscriptional (for review see [50]). Several RNA processing factors are recruited on the C-terminal domain of RNAPII and are deposited on the nascent pre-mRNA molecule during transcription elongation. Moreover, AS is influenced by the rate of transcription elongation; slowing down the polymerase may favour the use of weak splice sites by delaying the synthesis of downstream splice sites, thus facilitating the recognition of suboptimal exons (for review see [51]). In the light of these considerations it is not surprising that genotoxic treatments have been reported to induce transcription-dependent splicing alterations.

Recent studies have uncovered how the processivity of RNAPII can influence the recognition of alternative exons [52]. Muñoz and colleagues showed that DNA damage induced by UV can directly modulate the activity of RNAPII during transcript elongation thereby affecting the selection of alternative exons [53]. The effect of UV and cisplatin treatment on AS was initially characterized on the EDI alternative cassette exon of the fibronectin gene. Both genotoxic treatments despite eliciting quite different types of DNA damage strongly stimulated the inclusion of the EDI exon. The effects induced by UV were independent of p53 since they were observed also in Hep3B cells that are considered to be p53 null. Moreover, they were not due to changes in the expression or intracellular localization of splicing factors known to regulate this splicing event. Interestingly, however, both UV and cisplatin induced the hyperphosphorylation of RNAPII's CTD leading to the inhibition of transcription elongation. Therefore, in this case genotoxic damage affects the kinetic coupling between transcription and splicing, thereby affecting cotranscriptional AS. Interestingly, UV does not generally affect the level of either gene expression or AS, but its effects are restricted to a subset of responsive genes. In addition, the effects on AS induced by DNA damage may depend on the specific type of damage-inducing treatment. Indeed, when doxorubicin was used in this same study it did not induce the same splicing changes observed upon UV treatment [53].

Several splicing-sensitive microarray studies have examined the effect of CPT on cotranscriptional AS [54–56]. CPT appears to reduce RNA polymerase elongation rate promoting predominantly exon inclusion [54, 55]. Interestingly, many AS events leading to exon inclusion result in the production of mRNAs containing premature stop codons (PTCs) that will undergo nonsense-mediated decay. Gene Ontology analysis of the functional categories associated with the AS indicated that CPT treatment appeared to affect transcription and splicing of RNA-binding proteins [54, 55]. These observations suggest that this may represent a mechanism that allows the cell to respond to genotoxic damage by rapidly adjusting the level of RNA processing factors to the level of transcription. 

A specific example of AS event that is affected by DNA damage is provided by the MDM2 gene. As mentioned previously, the MDM2 gene produces several different mRNA variants due to AS. The biological significance of these variants is presently unknown since only few of them are translated into proteins. The best characterized alternative MDM2 mRNA isoform is the ALT1 transcript that lacks 8 of 12 exons due to exon skipping. It has been reported that the ALT1 variant is upregulated by UV treatment [12, 57]. Dutertre and colleagues demonstrated that production of ALT1 and other variants due to exon skipping is regulated cotranscriptionally [56]. Specifically, different genotoxic treatments such as camptothecin, doxorubicin, and cisplatin induce MDM2 exon skipping by disrupting the interaction between EWS, a transcriptional coregulator [58], and the splicing factor YB-1 [56]. These results suggest that DNA damage may interfere with the coupling between transcription and splicing leading to the production of alternative or aberrant mRNA variants.

Recent work that examined the response to IR using exon sensitive microarrays in lymphoblastoid cells and in fibroblasts confirms the genome-wide effects on transcription and splicing induced by genotoxic stress. Exon-level analysis revealed a general increase in internal exon skipping in response to radiation. The affected genes are involved in cell cycle regulation, chromatin dynamics, p53 regulation, and cell growth. In addition this study revealed an increase in the use of alternative promoters. These promoters have p53 binding elements at or near the start site suggesting that the protein isoforms encoded by these mRNA variants may have an active role in regulating the response to IR [59].

Collectively, these reports suggest that genotoxic damage can interfere with RNA polymerase II activity and may influence cotranscriptional AS by distinct mechanisms. However, considering that the functional categories associated with the affected genes are related to crucial cellular programs one can speculate that these changes might be functionally relevant for the response to DNA damage.

## 3. How AS Can Affect the DDR

**Figure 2:**
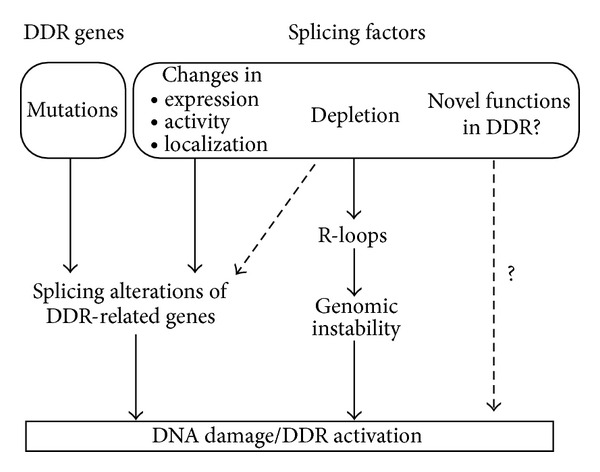
Alternative splicing alterations can activate the DDR. Mutations in splicing regulatory signals can inactivate the function of genes directly involved in the DDR, resulting in the accumulation of DNA damage. However, also the inactivation of canonical splicing factors can have similar effects, either by inducing aberrant splicing of DDR genes or by perturbing cotranscriptional splicing and inducing R-loop formation. However, it cannot be ruled out that AS- and RNA-binding proteins may play novel roles in the DDR and the control of genome stability.

Regulation of AS depends, on one hand, on the presence of specific sequence elements in the pre-mRNA and, on the other hand, on *trans*-acting protein factors. In this section we first report the most recent findings on how mutations in genes involved in the DDR affect splicing of the transcript thereby altering protein function. Then, we review how alteration of the expression of splicing factors can contribute to genomic instability and cancer (Figure 2). 

### 3.1. Mutations in Genes Involved in the DDR Can Disrupt Pre-mRNA Splicing

The splicing machinery assembles on conserved sequence elements that define the intron-exon junctions, the so-called splice sites, and on the branch point sequence (BPS), a poorly conserved sequence located near the 3′ end of the intron. In addition to these core splicing signals, splicing is influenced by other regulatory elements [60]. These elements are conventionally classified as exonic splicing enhancers (ESEs) or silencers (ESSs) depending whether they function to promote or inhibit inclusion of the exon they reside in and as intronic splicing enhancers (ISEs) or silencers (ISSs) if they enhance or inhibit usage of adjacent splice sites or exons from an intronic location. These regulatory elements function by recruiting factors that activate or inhibit splice site recognition or spliceosome assembly. 

Considering that about the 95% of our genes produce at least two isoforms [61] there is a high probability that mutations affecting these *cis-*acting elements could induce aberrant splicing with deleterious consequences. Accordingly, it has widely been suggested that most of the unclassified mutations (missense or silent) could affect splicing [62–65]. Furthermore, synonymous mutations, that do not change the protein sequence and therefore have traditionally been considered innocuous polymorphisms, could induce splicing aberrations by modifying splice sites (either canonical or cryptic) or splicing regulatory sequences [63, 64]. Splicing affecting mutations could lead to transcript instability by nonsense mediate decay (NMD) or to the synthesis of truncated or dysfunctional protein products. Despite their potential functional relevance, characterization of splicing-affecting mutations, in general, and in DDR genes, in particular, is not extensive. This may be in part due to historical reasons and also to technical issues related to the experimental validation of the functional consequences of a specific mutation. Hereafter, we describe examples of these types of mutations affecting critical genes in the DDR (see also Table 1). First, we will discuss mutations in the well-characterized BRCA1 and BRCA2 genes. Second, we will describe an example of intronic mutation affecting AS of the ATM gene. Then, we will illustrate how different AS isoforms of key DDR regulator can have different functions. Finally, we will review how the loss of splicing factors can cause DNA damage.

#### 3.1.1. BRCA1 and BRCA2: The Reclassification Issue

BRCA1 (OMIM 113705) and BRCA2 (OMIM 600185), the two most important breast cancer susceptibility genes [87], are key players in DDR. They are involved in homologous recombination (HR) and DNA repair (or review see [1]). 

More than 3500 mutations have been reported that affect the BRCA1/2 genes, about one-third of which are unclassified variants (UVs) (Breast Cancer Information Core Database (BIC), French UMD-BRCA1/2 mutation database: http://www.umd.be/BRCA2/, http://www.umd.be/BRCA1/) that may induce aberrant splicing. In 1998 Mazoyer et al. described a missense mutation within the exon 18 of the BRCA1 gene, which leads to exon skipping and consequently to the disruption of the BRCT domain, producing a nonfunctional protein [62]. Using an *in vitro* splicing system Liu and colleagues subsequently showed that exon skipping resulted from the disruption of a splicing enhancer in exon 18 [88]. Subsequently, Fackenthal et al. detected a missense mutation in BRCA2 affecting an ESE that caused the skipping of exon 18 and the out-of-frame fusion of exons 17 and 19 [68]. Later on, several other mutations that affect splicing were described in BRCA1 and BRCA2 contributing to a better understanding of the mechanism underlying the role of these proteins in tumorigenesis [65, 66]. More recently, Gaildrat et al. used a BRCA2 minigene reporter system to study the effect of predicted splice-site mutation and some unclassified mutations occurring at a distance from the splicing sites. The study identified a group of mutations that induced the aberrant splicing of exon 7 [69].

In recent years, several groups have proposed a combined approach to study the potential effects of BRCA1/2 genetic variants on splicing efficiency [64, 67]. These strategies exploit splicing prediction programs to detect potential splicing alterations followed by functional assays to analyze BRCA1/2 unclassified mutations. Using this approach Sanz and colleagues identified 57 putative splicing-affecting mutations. However, an effect on splicing could be confirmed by functional analysis only for half of the tested mutations [64]. This low rate of correlation could be explained by the high false positive outputs of the algorithm predicting ESE and ESS mutations. Therefore, the results of this study and of other similar reports underlie the importance of a functional validation of bioinformatic predictions [64, 67]. Nevertheless, these studies support the reclassification of many UVs as splicing affecting mutations.

#### 3.1.2. ATM: The Intronic Case

The ataxia-telangiectasia mutated (ATM) (OMIM 607585) gene, a key player in the DDR, is mutated in the autosomal recessive disorder ataxia-telangiectasia (AT, OMIN number 208900). The gene was identified in 1995 by positional cloning; it encodes 66 exons spanning ~160 kb of genomic DNA [89]. It soon became clear that a significant proportion of the known mutations in the ATM gene causes splicing defects [89–91]. Here, we will focus our attention on a specific aberrant splicing event that illustrates an additional layer of complexity in splicing regulation. 

The aberrant inclusion of a pseudoexon of 65 nt located in intron 20 (termed here exon 20A) was reported more than 10 years ago in a patient affected by AT [92]. The inclusion of exon 20A was associated with a deletion of four nucleotides (GTAA) in intron 20. The authors identified a novel regulatory element within intron 20, termed “intron-splicing processing element” (ISPE), which acts as an intronic silencer of a cryptic 3′ splice site [93–95]. The ISPE is recognized by the U1 snRNP, a core component of the splicing apparatus that normally binds the 5′ splice site. Sequestration of U1snRNP by the ISPE prevents inclusion of exon 20A. However, in the AT patient the four nucleotides deletion impairs U1 snRNP binding to the regulatory element, causing the inclusion of the cryptic exon [94]. 

### 3.2. Same Gene, Two Isoforms with Quite Different Functions

Cyclin D1 was first characterized as a cell cycle regulator. Cyclin D1 associates with CDK4/6 promoting the phosphorylation of Rb; this last event leads to the derepression of E2F, a transcription factor that controls the expression of DNA replication genes, allowing cell cycle progression (for review see [96]). In addition, cyclin D1 has a transcriptional regulatory activity that is independent of its association with CDK4/6 [96].

Two AS variants of cyclin D1 have been described, called D1a and D1b [97]. The respective protein isoforms display different intracellular localization: while D1a shuttles between the nucleus and the cytoplasm, D1b is constitutively nuclear [98]. Both transcript variants are expressed in normal tissues but D1b often appears upregulated in several forms of cancer, including breast cancer [99, 100]. The oncogenic properties of D1b isoform have been extensively described [98, 101]. Instead, cyclin D1a has directly been related to the DDR [102]. Recruitment of cyclin D1a, but not of D1b, to chromatin is sufficient to activate the DDR promoting H2AX phosphorylation. Moreover, after genotoxic stress D1a enhances the recruitment of repair factors contributing to checkpoint activation and G2/M arrest [102]. A recent report by Myklebust and colleagues identifies high expression of cyclin D1a protein as a positive predictive factor for the benefit of adjuvant chemotherapy with levamisole and 5-fluorouracil (5-FU), which leads to replication stress, due to the depletion of the intracellular deoxythymidine triphosphate (dTTP) pool in colorectal cancer [103]. This last report underlies the importance to link expression patterns of splicing isoforms with therapeutic approaches.

Two recent reports show that the splicing factors Sam68 and ASF/SF2 regulate cyclin D1 splicing favoring the D1b isoform [104, 105]. In addition, transcriptional regulation also affects the cyclin D1a/D1b ratio. Sanchez and colleagues reported that the EWS-FL11 fusion protein, a well characterized transcription factor, is able to favor the expression of the D1b isoform by decreasing the rate of polymerase II elongation [106]. Additionally, a polymorphism at the 3′ splice site of intron 4 may influence splice site choice favoring the production of the D1b isoform [97]. All these mechanisms show that alternative splicing of cyclin D1 is under a tight control making it a very interesting target for the development of new therapeutic strategies.

Another quite interesting example of AS variants was recently described by Pabla and colleagues [107]. CHK1-S is a novel splice variant of CHK1, which functions as endogenous regulator of CHK1 in both physiological conditions and after DNA damage. The authors demonstrated that in normal conditions CHK1-S is able to interact with CHK1 inhibiting its activity and promoting the S to G2/M transition. Upon DDR activation, CHK1 becomes phosphorylated and the CHK1-S/CHK1 interaction is disrupted, so that CHK1 can induce cell cycle arrest facilitating DNA repair. Interestingly, the authors reported a deregulated expression of CHK1-S in testicular and ovarian cancer. How the CHK1-S expression is regulated remains to be explored. 

### 3.3. Loss of Splicing Factors and Genomic Instability

A growing body of evidence suggests that the depletion of splicing factors may induce genomic instability. It is by now well established that transcription and RNA processing are tightly coupled processes [13, 14]. This, on one hand, favors accurate and efficient mRNA processing, and on one hand, protects the genome from the likely disastrous effects of the nascent transcripts themselves [108]. Accordingly, in *S. cerevisiae*, when genes involved in mRNA processing are mutated, defects occur in the packaging of nascent mRNAs [109]; as a result the nascent pre-mRNA hybridizes with the transcribed strand generating an RNA-DNA duplex, known as R-loop, causing genomic instability. Analogous effects have been observed in chicken DT40 cells upon silencing of the splicing factor ASF/SF2 [108, 110]. Similarly, in mouse embryonic fibroblasts, loss of SC35 (SFSR2) resulted in G2/M cell-cycle arrest and genomic instability [111]. Indeed, not only the siRNA-mediated silencing of splicing factors but also the genetic depletion of a transcriptional coactivator, such as SKIIP, besides affecting splicing, induced genomic instability [112]. Interestingly, overexpression of RNAse H, which cleaves the RNA molecule in a RNA-DNA hybrid, reduced the formation of H2AX foci [112]. Although the precise mechanism through which the formation of R-loops results in genomic instability is still unclear, these structures have recently been demonstrated to impair the replication fork progression [113]. 

Finally, in the last few years numerous mutations affecting components of the splicing machinery have been reported in several types of cancer [114–119]. The functional consequences of these mutations, and how they contribute to tumorigenesis not fully understood. However considering the genotoxic consequences of splicing factor depletion, discussed previously, it is possible that at least some of these mutations may result in the functional inactivation of the splicing factors thus affecting genomic stability. 

### 3.4. Splicing-Related Proteins as Novel Factors in the DDR

The results of several recent genome-wide studies strongly suggest an overlap between the pathways leading to mRNA biogenesis and the cellular response to DNA damage. Some years ago, Matsuoka and colleagues performed a large-scale proteomic analysis that identified more than 700 proteins phosphorylated by ATM and ATR in response to DNA damage. Interestingly, RNA processing was among the enriched Gene Ontology categories that had not been previously linked to the DDR [120]. The involvement of RNA maturation in the cellular response to DNA damage has been subsequently supported by various siRNA-based screens to identify genes involved in DNA damage sensitivity and genome stabilization [112, 121]. In particular, two genome-wide shRNA screens identified the Ewing sarcoma (EWS) protein, a member of the TET (TLS/EWS/TAF15) family of RNA- and DNA-binding proteins, as necessary for resistance to camptothecin [122] and IR [121]. Consistent with a role in the DDR, EWS knock-out mice show hypersensitivity to ionizing radiation [123]. Recently, Paronetto et al. showed that the Ewing sarcoma (EWS) protein, a member of the TET (TLS/EWS/TAF15) family of RNA- and DNA-binding proteins, regulates AS in response to DNA damage [124].

Using a similar approach to identify novel genes contributing to telomeres protection Lackner and colleagues observed that silencing of several splicing-related genes (including SKIIP and SF3A1) induced a general activation of the DDR, possibly because of R-loop formation while having a weak effect on telomere stability [125]. More recently, RBMX, an hnRNP that associates with the spliceosome and influences alternative splicing [126], was found in a genome-wide siRNA-based screen to detect regulators of homologous recombination (HR) regulators. RBMX regulates HR in a positive manner, accumulates at sites of DNA damage in a poly(ADP-ribose) polymerase 1- (PARP1-) dependent manner and promotes resistance to several DNA damaging agents [127]. As discussed in Section 2.2, Beli and colleagues identified the protein phosphatase PPM1G, that regulates spliceosome activity, using high resolution mass spectrometry combined with stable isotope labeling with amino acids in cell culture (SILAC) to quantify regulated changes in phosphorylation and acetylation induced by different DNA-damaging agents [28]. In addition, they detected the phosphorylation of THRAP3, a protein involved in RNA processing and stability [128, 129]. Interestingly, while many DDR factors are recruited to H2AX foci, THRAP3 is excluded from sites of DNA damage in a manner that parallels transcription inhibition. Two THRAP3-binding proteins BCLAF1 and PNN [130], that have also been implicated in mRNA metabolism, behaved in a similar manner suggesting that they are part of a novel protein complex, which may link RNA splicing to the DDR. A comprehensive review, that appeared while this paper was under revision, describes in detail some of these novel RNA-binding proteins involved in DDR [9].

## 4. Summary and Future Perspectives

Genomic instability is a hallmark of cancer cells, and the understanding of the mechanisms able to limit or counteract it can positively impact on the therapy of tumors. Genome-wide approaches have revealed that genes involved in RNA processing are often deregulated in response to genotoxic treatments. Since many chemotherapeutic compounds are DNA-damaging agents, AS can be an important determinant of how tumor cells respond to therapy.

Pre-mRNA splicing is a crucial step in the control of gene expression. The activity of splicing factors must therefore be tightly regulated since both their depletion and their upregulation can have harmful consequences. On one hand, deregulation of splicing factors may affect AS leading to the generation of cancer driving transcripts [131, 132]. On the other hand, depletion of splicing factors may induce aberrant splicing of critical DDR effectors altering indirectly the cellular response to DNA damage [133]. Moreover, splicing factors' depletion may slow down intron removal favoring the formation of DNA/RNA hybrid thereby leading to the collapse of replication forks and to the generation of DSBs [108, 112, 113]. Finally, the activation of the DDR can promote the posttranslational modification of splicing factors altering their intracellular localization and/or their activity [22, 23, 41, 46, 134]. Although some aspects of the relationship between DDR and mRNA processing have been clarified, certain observations are still to be explained. Why are some splicing factors recruited to sites of DNA damage? What are their functions there? Moreover, when splicing changes are considered, a causal link between splicing alteration and disease occurrence can only be established if the stability of the mRNA variant and the function of the encoded protein have been determined. Finally, the possibility should be considered that an involvement of splicing factors in the DDR may not necessarily imply an involvement of splicing regulation in the DDR but may reflect other functions of these proteins. Nevertheless, considering that AS plays a major role in the regulation of the apoptotic response (see C. Sette's review in this issue) and that AS variants have been demonstrated to regulate chemoresistance [103], it is reasonable that splicing modulation has been proposed as an appealing therapeutic target [135, 136]. Strategies to modulate AS by antisense oligonucleotides are already in advanced clinical trial phases for some neuromuscular disorders, such as Duchenne muscular dystrophy or spinal muscular atrophy, and oligonucleotides are being developed to target specific mRNA variants to enhance the efficacy of conventional chemotherapy [137]. In addition, in recent years several bacterial compounds, and other small molecules have been identified that target spliceosomal components [138]. Interestingly, some of these compounds display strong cytostatic effects and significant antitumor activity in animal models. Further understanding of how AS regulation and the DDR are interconnected and linked to different signal transduction pathways should help us to better understand tumor progression and provide a basis for innovative splicing-targeted cancer therapies. 

## Figures and Tables

**Table 1:** List of DDR-related genes found to be aberrantly spliced in several cancer types.

Gene	Function	Cancer	References
BRCA1	An E3 ubiquitin ligase contained in several cellular complexes, involved in DNA repair, genome stability maintenance, and cell cycle checkpoint control.	Breast and ovarian cancer	[62, 64–67]

BRCA2	Involved in HR, it associates with RAD51	Breast and ovarian cancer Familial pancreatic cancer	[64, 67–70]

ATM	Apical kinase of DDR response, mainly involved in HR	Ataxia-telangiectasia* Hereditary breast and ovarian cancer Mantle cell lymphoma Colon tumor derived cell lines Leukemia-lymphoma-derived cell lines	[71–74]

MRE11	Component of DNA damage sensor complex MRN	Mismatch repair deficient colorectal cancer Leukemia-lymphoma and colorectal cancer-derived cell lines	[73, 75]

ATR	Apical kinase of DDR response, mainly involved in HR	Seckel syndrome* Hodgkin's lymphoma Breast and ovarian cancer	[76–79]

XPA	Nucleotide excision repair	Xeroderma pigmentosum*	[80]

DNAPK	Apical kinase of DDR response, mainly involved in NHEJ	Xeroderma pigmentosum*	[81]

MSH2 and MLH1	Mismatch repair	Hereditary nonpolyposis colorectal cancer	[82–84]

CHEK2	DNA damage checkpoint kinase	Breast cancer Li-Fraumeni syndrome	[85, 86]

HR: homologous recombination; NHEJ: nonhomologous end joining. *ataxia-telangiectasia, xeroderma pigmentosum, and Seckel syndrome were included because they display strong predisposition to malignancies.
